# An update on the ornamental fish industry in Malaysia: *Aeromonas hydrophila*-associated disease and its treatment control

**DOI:** 10.14202/vetworld.2021.1143-1152

**Published:** 2021-05-11

**Authors:** Norashikin Anjur, Siti Fatimah Sabran, Hassan Mohd Daud, Nor Zalina Othman

**Affiliations:** 1 Department of Technology and Natural Resources, Faculty of Applied Sciences and Technology, Universiti Tun Hussein Onn Malaysia, Pagoh, Johor, Malaysia; 2 Department of Agrotechnology and Bio-industry, Politeknik Sandakan, Sandakan, Sabah, Malaysia; 3 Centre of Research for Sustainable Uses of Natural Resources, Universiti Tun Hussein Onn Malaysia, Pagoh, Johor, Malaysia; 4 Department of Veterinary Clinical Studies, Faculty of Veterinary Medicine, Universiti Putra Malaysia, Serdang, Selangor, Malaysia; 5 Aquatic Animal Health and Therapeutics Laboratory (AquaHealth), Institute of Bioscience, Universiti Putra Malaysia, Serdang, Selangor, Malaysia; 6 Innovation Centre in Agritechnology for Advanced BioProcess, Universiti Teknologi Malaysia, Pagoh, Johor, Malaysia

**Keywords:** *Aeromonas hydrophila*, chemotherapeutic drug, Malaysia, ornamental fish, phytotherapy

## Abstract

Malaysia is the world’s major producer and exporter of ornamental fish, contributing 9% to the global trade and taking the second position after Singapore. Because of their artistic appeal and tremendous commercial value for international trade, ornamental fish recently gain rapid importance for foreign exchange and as a source of employment. While ornamental fish production is growing, there is an increase in infectious diseases, resulting in high fish mortality with significant economic loss. Bacterial disease is a serious problem for ornamental fish industry. Bacterial species surveillance in diseased freshwater ornamental fish from an aquarium shop reveals that *Aeromonas hydrophila* is the most dominant bacteria isolated. Consequently, Malaysia is stepping up its efforts by implementing the Economic Transformation Program and other biosecurity steps to address the aquaculture issues and encourage the regrowth of the ornamental fish market. Chemotherapeutic medications, phytobiotics, probiotics, yeast extracts, vaccines, and disinfectants can be used in controlling bacteria. Further studies should be done to find new antibacterial agents from natural sources to combat bacterial fish diseases and reduce fish mortality rate in sustainable aquaculture farms. This review summarizes the literature on ornamental fish industries and aquaculture production in relation to *A. hydrophila*-associated diseases and ornamental fish health management in Malaysia.

## Introduction

Aquaculture production has developed rapidly since the early 1980s. Aquaculture became very important due to the high demand for fish and seafood products. Ornamental fish also contribute to the development of the aquaculture industry. Since the aquaculture activities of all aquatic organisms are the same, all management in every production level should be standardized to maintain sustainability in developing this industry. Asia is the primary aquaculture producer, contributing almost 90% of the world’s aquaculture production [1].

Ornamental fish are reared or kept not for any edible or angling qualities but for their visual appeal. About 1000 years ago, ornamental fish breeding and keeping started. Nowadays, many fish species are valued for their beauty and distinct markings [2]. While ornamental fish production is increasing due to high demand worldwide, it has resulted in increased infectious diseases, high fish mortality rate, and substantial economic losses. A significant problem in ornamental fish industry is the *Aeromonas*-related bacterial disease. Bacterial infections associated with motile *Aeromonas hydrophila* were reported in 14 ornamental fish species [3]. *A. hydrophila* was also identified as the causative agent of the disease outbreak in goldfish, *Carassius auratus*, from four ornamental fish farms in Kerala, India [4].

Bacterial species surveillance in diseased freshwater ornamental fish from an aquarium shop in Kuala Terengganu, Malaysia, reveals that *A. hydrophila* is the most dominant bacteria isolated. Consequently, Malaysia is stepping up its efforts by implementing the Economic Transformation Program and other biosecurity steps to address the aquaculture issues and encourage the regrowth of the sustainable ornamental fish market [5]. The worldwide development of ornamental fish trade favors the wide-spreading of their pathogens, such as bacteria, parasites, fungi, and viruses, which are often transported with fish and should be focused on to sustain the ornamental fish industry [3].

Thus, this current review summarizes the literature on ornamental fish industries and aquaculture production in relation to *A. hydrophila*-associated disease and ornamental fish health management in Malaysia.

## Global Ornamental Fish Industry

Ornamental fish is the most popular pet in the United Kingdom (UK), with more than 100 million pet fish kept in aquariums and ponds. Around 4 million households own a pet fish in UK, which is 14% of their population. UK fish keepers spend about £400 million a year on their hobby. About 3000 pet shops operate in UK, and 2/3 from them are selling pet fish. Pets are also good for our health. It is estimated that £2.45 billion amount of fish save the National Health Service every year. Watching fish in an aquarium reduce blood pressure and anxiety. Furthermore, having an aquarium in a home’s dining room led to residents having a better appetite [6].

The United States of America (USA) and European Union (EU) are reported to be the top global importers of aquarium fish, followed by UK and Singapore, the leading fish aggregator and exporter in Asia [6]. Due to their artistic appeal and immense commercial interest for international trade, ornamental fish recently gain rapid importance in earning foreign exchange and as a source of employment. The art of maintaining an aquarium is ancient, and it started about 100 years ago with the goldfish, *C. auratus*, a globally popular ornamental fish. The appealing color and calm temperament of ornamental fish offer people a source of pleasure and happiness, regardless of their age [7].

Ornamental fish also contribute to import and export activities and foreign exchange because of its popularity as an easy and stress-relieving hobby. About 7.2 million houses in USA and 3.2 million in EU have an aquarium, and the number is increasing daily worldwide. Ornamental fish farming is developed to fulfill the demand. USA, Europe, and Japan are the largest markets for ornamental fish globally, but more than 65% of the exports come from Asia. This will encourage economic development [8].

## Malaysia Ornamental Fish Industry

In recent years, ornamental fish production is recorded as 325 million pieces for RM 350 million. The major groups of ornamental fish produced in Malaysia consist of freshwater species from the family of Anabantidae, Callichthyidae, Characidae, Cichlidae, Cyprinidae, Cyprinodontidae, Loricariidae, Osteoglossidae, and Poecilidae [9]. The values of ornamental fish imported and exported are RM 4,342,143,046 and RM 3,157,650,332, respectively [10]. At present, there are 229 registered ornamental fish exporters in Malaysia. The main ornamental fish exporter is from the state of Johor (86), followed by Perak (49), Selangor (38), and Penang (35). Others are from Kuala Lumpur (8), Negeri Sembilan (6), Melaka (3), Kedah (2), and Sarawak (2), whereas the main ornamental fish importer is from Selangor (52), Johor (29), Penang (29), and Perak (25). Others are from Kuala Lumpur (12), Melaka (6), Negeri Sembilan (6), Kedah (2), Terengganu (2), and Sarawak (1) [11].

*Xiphophorus hellerii*, *Xiphophorus maculatus*, *Poecilia sphenops*, and *Trichopodus trichopterus* serve as the important species for ornamental fish trade in Malaysia [12]. The European Ornamental Fish Import and Exports reported that Malaysia had recorded an income of $1,133 million through ornamental fish exportation to the EU countries [13]. The export markets covered by Malaysia’s ornamental fish industry are more than 30 countries, including UK, US, Germany, Italy, Hong Kong, Spain, Japan, and Taiwan. The top three leading exported species are golden arowana (*Scleropages formosus*), various goldfish, and discus [14]. This ornamental fish industry is very important, with 548 employers and 1433 employees in Malaysia. This will improve the economic level of the population and nation [15].

Recent reports from the Department of Fisheries presented that Malaysia is the 8^th^ largest world producer of ornamental fish, and more than 70% of ornamental species produced are exported. Malaysia contributes 9% to the global trade and holds the second position after Singapore [14]. As a growing industry, current practices have led to disease outbreaks which are highlighted as one of the major issues faced in aquaculture farms. In 20 years of disease reporting in Malaysia, several bacterial and viral diseases were found to persist in farms. In addition, emerging global diseases have also been detected in several farms. These disease outbreaks led to huge economic losses. Eventually, the combination of persistent and emerging diseases creates a potential threat to the aquaculture industry; hence, immediate attention is required [16].

## Ornamental Fish Health Management

Fish are susceptible to the same types of pathogens that affect warm-blooded animals, including bacteria, viruses, fungi, parasites, or a combination of these pathogens, and various noninfectious agents. Among these, bacterial fish diseases are considered to be the major problem in the aquaculture industry [17]. Some diseases do not show non-specific clinical signs, such as pale gills, enlarged liver, and distended body [12]. For example, infectious spleen and kidney necrosis virus-infected fish from four different families were asymptomatic in major ornamental fish breeding states in Peninsular Malaysia. They appeared clinically healthy [18]. Some infested fish show behavioral abnormalities, including irritation, discoloration, lethargy, and anorexia. The pathogen invades various host organs and releases cytolytic toxins. Other pathogenic symptoms include red fin disease and hemorrhagic septicemia [7].

Nonetheless, most diseases can be avoided with proper management. Daily fish health observation is very important in fish keeping and culture. To facilitate the daily evaluation of fish welfare to avoid stress, several simple indicators to be observed have been proposed, such as color, ventilation rate, swimming pattern and other behaviors, food intake, growth rate, condition, presence of morphological abnormalities, injury, and reproductive performance [19]. It should be noted that these indicators should also be a part of the routine diagnostic workup and daily assessment of the fish population.

This health management practice is also recognized as one of the important criteria for any farm to be certified with the Malaysian Good Agricultural Practices (myGAP) certificate. Certified aquaculture farms have to fully comply with at least 19 certificate requirements from site selection, wastewater treatment, hygiene practices, animal health, halal, etc., before the certificate can be awarded. This myGAP certification is managed by the Fisheries Biosecurity Division, Department of Fisheries Malaysia [20,21]. All the criteria and guidelines for good aquaculture practice were documented in the e-book titled “Kit Akuakultur Baik” of the Department of Fisheries Malaysia [22].

The Department of Fisheries Malaysia is the custodian of the 1985 Fisheries Act, which serves as the principal legislative source for subsidiary regulations, including aquaculture and fisheries health management. Legislation passed by other government entities also plays a significant role in governing operations which can directly or indirectly affect Malaysia’s aquaculture and fish safety. The Department of Fisheries Malaysia, Malaysia Quarantine and Inspection Services, and Department of Veterinary Services collaborate in fish health management [20].

The factors that could contribute to bacterial infection in ornamental fish include poor water quality, crowding, transportation, and inadequate nutrition [5]. The health and nutrition of ornamental fish are of paramount importance in ornamental fish trade. To keep the fish in healthy condition, maintenance of suitable water quality greatly reduces the various stressors where fish are exposed to, reducing the likelihood of diseases. The frequency of water quality monitoring depends on the type of production systems and specific parameters being monitored. Other than that, fish will remain healthy and grow rapidly if high-quality feeds with required nutrients are provided. Fish that are fed with nutritionally complete diet are more capable to cope with stress and resist diseases. Light, noise, and other disturbances can also stress fish and should be minimized [23].

## *A. hydrophila*-associated Disease

Ornamental fish can suffer from different bacterial diseases. The most prevalent infections are caused by *Aeromonas*, *Shewanella*, *Citrobacter*, *Plesiomonas*, *Edwardsiella*, and *Pseudomonas* species. There is also a broad spectrum of rarely identified bacteria which may be the causative agents of diseases [3]. *A. hydrophila* is one of the common freshwater pathogens [24]; it leads to a great economic loss in aquaculture [25]. It has evolved in a wide range of temperatures, conductivities, pHs, and turbidities, and only few environments with extreme ranges of these parameters (extremely saline conditions, hot springs, and highly polluted waters) fail to develop aeromonads [26]. It can be found in fish farms, culture tanks, and aquaculture environments, such as water column and bottom sediment [27].

*A. hydrophila* is an opportunistic pathogen that is associated with various diseases in ornamental fish under stress [28]. This bacterium can attack aquaculture-raised species and wild fisheries. *A. hydrophila* produces some pathogenic factors, and the most important among them are hemolysin and aerolysin, leading to disease. The most common disease caused by *A. hydrophila* is motile *Aeromonas* septicemia (MAS). Freshwater and saltwater fish species are susceptible to this disease. The disease is manifested clinically with hemorrhages, ulcerations, abscesses, ascites, and anemia. Mortality rates are high, and aquaculturists incur substantial economic losses, thereby necessitating timely measures for disease control, prevention, and treatment [29].

Despite *Aeromonas* being commonly reported as the major cause of mortality in the industry, only two cases of outbreaks were reported in Malaysia. The first case of mass mortality reported in a tilapia farm identified the causal agents as a combination of *A. veronii* and Tilapia Lake Virus [30]. The second case reported the isolation and identification of *A. hydrophila* in diseased catfishes from a local farm, displaying the common clinical and histological symptoms of MAS [31]. Both cases were from food fish farms. At present, there is no reported mass mortality caused by *Aeromonas* infection in the ornamental fish industry of Malaysia. In India, the mass mortality of *C. auratus* was reported to be caused by *A. hydrophila* infection [4].

A survey on bacterial diseases in a retail pet shop of freshwater ornamental fish in Kuala Terengganu, Terengganu, and Malaysia was conducted from July to September 2007. Fifty diseased freshwater ornamental fish were collected from an aquarium shop in Kuala Terengganu. There were 26 isolates examined for suspected single and pure bacterial colony from diseased fish. Among the 26 strains inoculated, 15 of them were *A. hydrophila*, and the others were *Chromobacterium violaceum* (3), *Acinetobacter lwoffii* (1), *Acinetobacter baumannii* (1), *Edwardsiella tarda* (1), *Enterobacter* spp. (1), *Flavobacterium* spp. (1), *Serratia marcescens* (1), *Stenotrophomonas maltophilia* (1), and *Yersinia* spp. (1). The most common isolated bacteria were *A. hydrophila* [5].

## Bacterial Disease Treatment

There are a lot of chemicals being used in aquaculture to keep fish in healthy condition. The chemicals used in aquaculture can be classified based on purpose – as antibiotics or antimicrobials, disinfectants, chemotherapeutic agents, piscicides, hormones, and anesthetics [32]. Bacterial diseases like MAS can be regulated in fish using chemotherapeutic drugs, phytobiotics, probiotics, yeast extracts, vaccinations, and disinfectants. Based on experience, chemotherapeutics are most widely used in fish afflicted with MAS [29]. *A. hydrophila* infection is mainly controlled by antibiotics, such as oxytetracycline (OTC), sulfadimethoxine, and ormetoprim [33]. Since *A. hydrophila* is an opportunistic pathogen, the avoidance of factors predisposing to this disease and compliance to routine sanitation procedures on fish farms can also be the best method for disease control and prevention [29].

## Chemotherapeutic Drug

Chemical treatment is conventionally applied because it is easier to get drug and medical supplies. These drugs and chemicals may be used as disinfectants, herbicides, pesticides, spawning aids, and vaccines for disease prevention. However, aquaculturists must have access to regulated and controlled chemicals that are safe and effective and apply them in a manner that is consistent with their intended use, best management practices, and relevant rules and regulations [32] because these drugs find their way to the local drainage system and eventually contaminate the rivers [34].

To prevent and regulate bacterial diseases, the use of chemotherapy drugs has also increased. The three major groups of commonly used chemotherapeutants are topical disinfectants, antimicrobials, and probiotics. There is a wide range of topical disinfectants used by aquafarmers. The most common of these include formalin, benzalkonium chloride, acriflavine, malachite green, hypochlorite, and polyvinylpyrrolidone. Of these, acriflavine and malachite green are only used in hatcheries, while the others are used in both ponds and hatchery systems [35]. At present, there is no reported usage of polyvinylpyrrolidone, acriflavine, and malachite green. Malachite green was also a banned disinfectant for aquaculture in all countries, including Malaysia [32].

**Figure-1:**
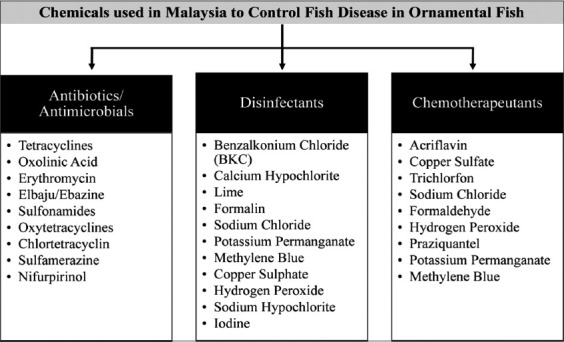
List of chemicals used for disease treatment and health management in Malaysia ornamental fish industry.

For exportation and marketing, ornamental fish are treated with some chemicals to keep them alive and to reduce stress and infection during transportation. During fish transportation, clinoptilolite and methylene blue are usually added into the water during the packaging process to remove biowaste ammonia and inhibit bacterial growth, respectively [34]. The other chemicals that are widely used in Malaysia’s ornamental fish industry are shown in Figure-1 [32].

Because of antibiotic overuse, drug-resistant strains are rising rapidly. In addition, the biofilms formed by this bacterium limited the antibacterial effect of antibiotics [25]. Diverse antibiotic-resistant bacteria, such as *Acinetobacter*, *Comamonas*, *Edwadsiella*, *Aeromonas*, and *Enterobacter*, and their implication on human health were reported from Malaysian aquaculture farms. *A. hydrophila* isolated from aquaculture pond and sediment shows antibiotic resistance against piperacillin, norfloxacin, and ceftazidime [27]. The higher multiple antibiotic-resistant indexes of pathogens and higher minimal inhibitory concentration of antibiotics for resistant isolates highlighted the excessive use of antibiotics in aquaculture farms [36].

An antibiotic sensitivity test was done for *A. hydrophila* isolates from diseased freshwater ornamental fish collected from an aquarium shop in Kuala Terengganu, Terengganu, and Malaysia. Fifteen *A. hydrophila* were isolated from eight species of ornamental fish. Seven types of antibiotics were used. The result shows that all *A. hydrophila* isolates are resistant to sulfamethoxazole. Thirteen of them are resistant to OTC, and eight of them are resistant to nalidixic acid. Six, two, and one isolates are resistant to furazolidone, chloramphenicol, and erythromycin, respectively. However, all isolates are sensitive to kanamycin [5]. This antibiotic resistance is also being studied in other countries, as shown in Table-1[28,36-42].

**Table-1:** Antibiotic-resistant study on bacterial isolates from ornamental fish in other countries.

Sample where isolates were taken	Findings	Country	Reference
Fish imported from Colombia, Singapore and Florida	Less resistant to Cefotaxime (16%), highest resistant to tetracycline (77%)	North America	[37]
Septicaemia freshwater ornamental fish from pet shop	Highest resistances towards tetracycline (58.5%) and erythromycin (54.7%)	Sri Lanka	[38]
Fantail, Angel fish, Koi carp, Molly, Swordtail and Guppy from ornamental fish shop	Resistant to ampicillin	Egypt	[39]
Zebrafish from pet shop	Resistant to amoxicillin (100%), nalidixic acid (100%), OTC (100%), ampicillin (93.02%), tetracycline (74.42%), rifampicin (67.44%) and imipenem (65.15%)	Seoul, Korea	[28]
Guppies from ornamental fish farm	Multidrug-resistant of *Aeromonas* spp. which comprise different antimicrobial resistance genes and Class 1 integrons	Seoul, Korea	[40]
Diseased freshwater ornamental guppy fishes from an ornamental fish farm	All of the isolates possessed the MAR index of greater than 0.2, indicating the heavier dose of antibiotics in the farm and the possibility of heavier dissemination of antimicrobial-resistant genes among the ornamental fish	Kochi, India	[41]
Naturally infected freshwater koi and goldfish	Show significant resistance pattern of isolates toward 47 antibiotics	Kochi, India	[36]
Ornamental fish imported from Singapore, Israel, Thailand, Sri Lanka, Czech Republic, Vietnam and Indonesia	Prevalence of antibiotic resistance in isolated bacteria was high (61%) compared to intermediate (11%) and sensitivity (28%) categories and varied across antibiotics. Bacteria showed resistance to lincomycin, ampicillin, OTC, and tetracycline, while sensitivity was found for florfenicol, chloramphenicol, gentamicin, and trimethoprim/sulfamethoxazole	Northwest Italy	[42]

OTC: Oxytetracycline

## Phytotherapeutic Agents

This problem on drug resistance leads to greater attention to seek for new antibacterial agents from natural sources to combat fish diseases in the aquaculture industry [43]. The past review by [44] critically evaluated the status of knowledge on phytotherapy against various aquacultural diseases in the world, identifying the bottlenecks and suggesting remedial measures. Phytotherapy becomes recognized as a handy and viable alternative to chemotherapy because it is economical, effective, non-resistance forming, renewable, eco-friendly, and farmer-friendly. A study on medicinal plants with antibacterial, antiviral, and antifungal activities is tabulated together with the herbs used as biopesticides and immunostimulants.

Medicinal plants are widely used as phytotherapeutic agents to treat infectious diseases in animals and humans since ancient times, and its therapeutic use for bacterial diseases in fish is safe. Medicinal plants can prevent diseases and restrict the emergence of *A. hydrophila* strains, and their beneficial effects are reflected by the enhancement in fish growth and resistance to MAS [29]. Furthermore, numerous investigations have pointed out the great antimicrobial potential of herbs as alternative biomedicine in aquaculture [45]. This review emphasizes its phytotherapeutic effect against aquaculture pathogenic bacteria in Malaysia (Table-2) [24,43,46-60].

**Table-2:** Phytotherapeutic study related to aquaculture pathogenic bacteria in Malaysia.

Isolates sources/host	Plants	Isolates	Method	Plant preparation	Findings	Reference
Tiger Shrimp (*Penaeus monodon*), Oyster (*Crassostrea iredalei*), and Red Tilapia (*Tilapia* spp.)	*Aloe vera*, *Colocasia esculenta*, *Citrus microcarpa*, *Centella asiatica*, *Ipomoea reptans*, *Morinda citrifolia*, *Murraya koenigii*, *Pandanus odoratissimus* and *Passiflora foetida*	*Vibrio alginolyticus I Vibrio parahaemolyticus*, *Vibrio harveyi*, *Vibrio vulnificus*, *Vibrio cholerae*, *Escherichia coli*, *Citrobacter freundii*, *E. tarda*, *Aeromonas hydrophilla*, *Salmonella* spp., *Schewanella putrifaciens* and *Streptococcus* spp.	*In vitro* (disk diffusion technique)	Aqueous and methanolic extracts	The most active antimicrobial plants were *Colocasia esculenta*, *Citrus microcarpa*, *Centella asiatica* and *Morinda citrifolia*	[46]
Tilapia fingerlings	Garlic, cinnamon, ginger, lemongrass, thyme, curry, mustard, turmeric, cubeb, clove	*Streptococcus agalactiae*	*In vivo* (diet and challenge)	Extracted with distilled water, mixed with fish feed	*Cinnamomum verum* extract displayed the highest antimicrobial activity	[47]
African catfish *Clarias gariepinus* (Bloch) fingerlings	Garlic peel	*A. hydrophila*	*In vivo* (diet and challenge)	Plant powder was incorporated into fish feed	Significantly higher survival rates were recorded in all the fish fed with garlic peel feed	[48]
Shrimps	*Colocasia esculenta*	*Vibrio alginolyticus*, *V. cholera*, *V. harveyi*, *V. parahaemolyticus* and *V. vulnificus*	*In vitro* (disk diffusion method)	Methanolic and aqueous extracts	*Vibrio* spp. showed sensitiveness to the extraction of *C. esculenta*, this plant could probably be used in prevention of vibriosis outbreak in shrimp farmings	[49]
Isolate from disease fish and test on Fingerling African catfish (*Clarias gariepinus*)	*Morus alba* (white mulberry)	*A. hydrophila*	*In vivo* (feed additive)	Methanolic extracts	Dietary supplements of the *Morus alba* foliage enhanced survival rate and treatment of the African catfish	[50]
Culture stock (1 freshwater, 4 marine)	Eight seaweed species and three seagrass species	*A. hydrophila*, *Vibrio harveyi*, *Vibrio harveyi*, *Vibrio alginolyticus*, *Vibrio parahaemolyticus* and *Vibrio anguillarum*	*In vitro* (disk diffusion method, MIC and MBC)	Methanolic extract	Most of the seaweeds and seagrass possess antibacterial activity against all the pathogen	[51]
Infected tilapia	*Cinnamomum camphora*, *Euphorbia hirta*, *Azadirachta indica*, *Carica papaya*	*Streptococcus agalactiae*	*In vivo* (diet and challenge)	Methanolic extracts and add to diet	Dietary *C. camphora* bark extract acted as a strong prophylactic to *S. agalactiae*	[52]
Diseased red hybrid tilapia	*Peperomia* *pellucida*	*A. hydrophila*	*In vivo* (diet and challenge)	Methanolic extracts coated onto fish pellet	*P. pellucida* leaf extract has potential in controlling motile septicaemia motile	[53]
Culture stock (common freshwater pathogen)	*Vitex trifolia*, *Aloevera*, *Strobilanthes crispus*, *Clinacanthus nutans*, *Pereskia grandifolia* and *Peperomia pellucida*	*Streptococcus agalactiae*, *A. hydrophila* and *Enterobacter cloacae*	*In vitro* (disk diffusion technique)	Aqueous and methanolic extracts	Strong antibacterial activity in *V. trifolia, A. vera* and *S. crispus* extracts	[24]
*Oreochromis* spp. Fingerling	*Vitex trifolia*, *Aloe vera* and *Strobilanthes crispus*	*Streptococcus agalactiae*	*In vivo* (challenge and diet)	Aqueous and methanolic extracts	Indicating improve defence system in the fish fed with *V*. *trifolia*, *S. crispus*, and *A. vera*	[54]
Diseased tilapia, *Oreochromis niloticus*	*Excoecaria agallocha*	*Streptococcus agalactiae*	*In vivo* (diet and challenge)	Methanolic extracts, dissolved in distilled water and sprayed on the thin layer of feed	Improve disease resistance	[55]
Culture stock	*Piper betle*	*Bacillus* spp., *E. faecalis*, *S. aureus*, *S. agalactiae*, *A. hydrophila*, *E. coli*, *K. pneumonia*, *P. aeruginosa*, *V. alginolyticus*	*In vitro* (TLC agar overlay bioautography assay)	Methanolic extracts	Successful antibacterial activity against several fish pathogenic bacteria	[56]
Asian sea bass, *Lates calcarifer*	*Piper betle*	*Vibrio alginolyticus*	*In vitro* (Disk diffusion method, MIC and MBC)	Ethanolic extract	Potential use of betel leaf crude extract as antimicrobial agent against marine bacteria	[57]
Culture stock obtained from Universiti Malaysia Terengganu	*Cosmos caudatus*	*A. hydrophila*	*In vitro* (disk diffusion assay and brine shrimp lethality bioassay)	Methanolic extract	All the tested concentrations inhibit the bacterial growth. The effective and safe level of *C. caudatus* extract is lower than 100 μg mL^−1^	[58]
Culture stock obtained from the Laboratory of Fish Health in the Aquaculture Department, Universiti Putra Malaysia	*Allium sativum* (clove and peel)	*A. hydrophila*, *Vibrio anguillarum*, *Vibrio alginolyticus*, *Vibrio harveyi.*	*In vitro* (disk diffusion method, MIC and MBC)	Aqueous and methanolic extracts	The study suggest that clove extract of *A. sativum* has the potential to be used as a phytobiotics in controlling the growth of marine pathogens	[59]
Bacterial Collection of the Aquatic Animal Health Unit, Universiti Putra Malaysia	*Polygonum chinense*, *Syzygium polyanthum*, *Premna foetida*, *Pimenta* *dioica*, *Brucea javanica*, *Vitex negundo*, *Alpinia conchigera* and *Clinacanthus nutans*	*Vibrio harveyi, Vibrio alginolyticus, Vibrio parahaemolyticus* and *A. hydrophila*	*In vitro* (disk diffusion method and brine shrimp cytotoxicity assays)	Methanolic and aqueous extracts	Extracts of *P. chinense* and *P. foetida* show high bactericidal activity and low toxicity could be a good potentials for use in fish culture	[43]
*Oreochromis niloticus* (red Nile tilapia)	*Terminalia catappa* Linn.	-	*In vivo* (static toxicity exposure)	Fine powder diluted in distilled water	The present study will be beneficial in considering the proper utilization of *T. catappa* leaf as potential antibacterial agent	[60]

A. hydrophila=*Aeromonas hydrophila*, E. tarda=*Edwardsiella tarda*

Plant extracts were reported to act as anti-stress, growth performance enhancer, appetite stimulator, and anti-pathogen in fish and shrimp aquaculture because plants contain alkaloids, terpenoids, tannins, saponins, glycerol, flavonoids, steroids, or essential oils. Some plants are rich in secondary metabolites and phytochemicals which have an effect on viral, bacterial, and parasitic fish diseases. Its main advantage is its natural origin, and most beneficial plants do not harm humans, fish, and the environment. The prevention of bacterial infections using plant extracts is highly desirable due to its low cost and high efficacy against certain bacteria compared to antibiotics that may harm the environment [61,62].

## Threat and Sustainability Issue

The potential risk of occurrence of new diseases associated with live animal trade is well-known [63]. Aquarium ornamental pet fish can be marketed and distributed from different areas and countries worldwide. Commercialization and circulation of live animals without the use of adequate prophylactic management procedures enable the dissemination of various agents responsible for infectious diseases [64]. In spite of these disadvantages, ornamental fish production still becomes more intense, and infectious diseases have increased, resulting in high fish mortality and significant economic loss [7].

At present, chemotherapy is the only option that is conventionally practiced for the prevention and treatment of aquaculture disease outbreaks. However, the use of chemical drugs has several negative impacts on the environment and humans. The use of most antibiotics is banned in aquaculture as it can develop drug-resistant bacterial strains and contribute to the unacceptable levels of antibiotic residues in fish tissues and the environment. Hence, the stimulation of non-specific immune system is a smart choice available to enhance the immunity and growth performance of cultured species. The wide usages of chemical drugs require further improvement in management to enhance the development of ornamental fish production [65-67].

The occurrence and incidence of antibiotic-resistant pathogens led to the ineffectiveness of most available antibiotics in the market for disease control [1,43]. Hence, in recent years, attention is given to eco-friendly and sustainable aquaculture disease management practices [66,67]. Malaysia is increasing its efforts by developing the Economic Transformation Program and several biosecurity measures to overcome these aquaculture issues and promote the regrowth of the ornamental fish industry [12]. It is necessary to implement optimal management and biosafety programs in production systems to achieve sustainable aquaculture in ecological, economic, and social terms [68].

The aquaculture sector is most often blamed for some irresponsible practices and biodiversity loss. Thus, greater conscious efforts must be done in managing aquaculture farms to ensure that biodiversity is conserved. The future activities of aquaculture industry would ensure reliable and sustainable aquaculture production. Environment-friendly feeds and culture consumption from regionally available ingredients should be developed. The establishment of technology management for aquaculture environment should consider the ecosystem approach to aquaculture. The impacts of the transfer and adoption of currently developed sustainability in aquaculture management should be accessed and analyzed. All these efforts focus on the development of environment-friendly-based aquaculture technologies [69].

## Conclusion

Malaysia needs more comprehensive mechanisms to monitor the types of chemotherapeutic agents used and how they are given. Studies on commonly used chemotherapeutic agents should be conducted in relation to the host’s residual patterns, and it is very important because of its health and environmental impacts. The insufficient knowledge of plant extracts limits its cost-effective use in ornamental fish aquaculture. Therefore, more studies are necessary to standardize plant extract administration methods. More studies are required to further validate the use of plant extracts in aquaculture, focusing on its growth-promoting effects, immune-stimulating effects, sex reversal effect, toxicity, extraction methods, and extract concentration, among others.

Even though the number of studies on medicinal plant application in aquacultures is increasing, more studies are needed to determine the exact mode of preparation, application, dosage, treatment duration, and effects of various medicinal plants on different fish species. Extensive research on phytochemicals and therapeutic agents is recommended, especially in using cheaper sources such as agriculture and food waste which possess medicinal value, so that more potential plants can be marketed and used in the aquaculture industry.

## Authors’ Contributions

SFS, HMD, NZO, and NA contributed to the conception of the specific review and collected literatures. NA contributed in the writing of the original draft, review and editing. SFS contributed to the review, editing, and supported in supervision. All authors read and approved the final manuscript.
